# Contact tracing of COVID-19 in Karnataka, India: Superspreading and determinants of infectiousness and symptomatic infection

**DOI:** 10.1371/journal.pone.0270789

**Published:** 2022-07-11

**Authors:** Mohak Gupta, Giridara G. Parameswaran, Manraj S. Sra, Rishika Mohanta, Devarsh Patel, Amulya Gupta, Bhavik Bansal, Vardhmaan Jain, Archisman Mazumder, Mehak Arora, Nishant Aggarwal, Tarun Bhatnagar, Jawaid Akhtar, Pankaj Pandey, Vasanthapuram Ravi, Giridhara R. Babu

**Affiliations:** 1 Cleveland Clinic Foundation, Cleveland, Ohio, United States of America; 2 All India Institute of Medical Sciences (AIIMS), New Delhi, India; 3 Indian Institute of Science Education and Research (IISER), Pune, India; 4 Beaumont Hospital, Royal Oak, Michigan, United States of America; 5 ICMR- National Institute of Epidemiology, Chennai, India; 6 Secretariat, Health & Family Welfare Services, Government of Karnataka, Bengaluru, India; 7 Office of Commissioner, Health, Family Welfare and AYUSH Services, Government of Karnataka, Bengaluru, India; 8 Department of Neurovirology, National Institute of Mental Health and Neurosciences (NIMHANS), Bengaluru, India; 9 Indian Institute of Public Health, Public Health Foundation of India, Bengaluru, India; The Chinese University of Hong Kong, HONG KONG

## Abstract

**Background:**

India has experienced the second largest outbreak of COVID-19 globally, yet there is a paucity of studies analysing contact tracing data in the region which can optimise public health interventions (PHI’s).

**Methods:**

We analysed contact tracing data from Karnataka, India between 9 March and 21 July 2020. We estimated metrics of transmission including the reproduction number (R), overdispersion (k), secondary attack rate (SAR), and serial interval. R and k were jointly estimated using a Bayesian Markov Chain Monte Carlo approach. We studied determinants of risk of further transmission and risk of being symptomatic using Poisson regression models.

**Findings:**

Up to 21 July 2020, we found 111 index cases that crossed the super-spreading threshold of ≥8 secondary cases. Among 956 confirmed traced cases, 8.7% of index cases had 14.4% of contacts but caused 80% of all secondary cases. Among 16715 contacts, overall SAR was 3.6% [95% CI, 3.4–3.9] and symptomatic cases were more infectious than asymptomatic cases (SAR 7.7% vs 2.0%; aRR 3.63 [3.04–4.34]). As compared to infectors aged 19–44 years, children were less infectious (aRR 0.21 [0.07–0.66] for 0–5 years and 0.47 [0.32–0.68] for 6–18 years). Infectors who were confirmed ≥4 days after symptom onset were associated with higher infectiousness (aRR 3.01 [2.11–4.31]). As compared to asymptomatic cases, symptomatic cases were 8.16 [3.29–20.24] times more likely to cause symptomatic infection in their secondary cases. Serial interval had a mean of 5.4 [4.4–6.4] days, and case fatality rate was 2.5% [2.4–2.7] which increased with age.

**Conclusion:**

We found significant heterogeneity in the individual-level transmissibility of SARS-CoV-2 which could not be explained by the degree of heterogeneity in the underlying number of contacts. To strengthen contact tracing in over-dispersed outbreaks, testing and tracing delays should be minimised and retrospective contact tracing should be implemented. Targeted measures to reduce potential superspreading events should be implemented. Interventions aimed at children might have a relatively small impact on reducing transmission owing to their low symptomaticity and infectivity. We propose that symptomatic cases could cause a snowballing effect on clinical severity and infectiousness across transmission generations; further studies are needed to confirm this finding.

## Introduction

COVID-19, a pneumonia caused by the novel coronavirus SARS-CoV-2 originated in Wuhan, China [1]. As of September 2021, the pandemic had spread to over 200 countries and territories, causing over 219M cases and 4.5M deaths of which India contributed to 33.6M cases with over 446,000 deaths [2]. Karnataka, a south-Indian state inhabited by more than 61 million people [3], detected its first COVID-19 case on 9th March 2020.

Contact tracing remains one of the key public health responses in infectious disease control with a history that can be traced back to the late 19th century [4]. In the present COVID-19 pandemic, contact tracing may achieve significant outbreak control when effective reproduction number is lower due to social distancing and public health interventions (PHI’s) [5]. In addition, primary contact tracing data is extremely valuable in elucidating transmission characteristics of an infectious disease which can be used optimise PHI’s.

A disease’s basic reproduction number R0 describes the ‘average’ number of secondary infections (offsprings) generated by a single infected individual. Super-spreader events (SSEs) highlight a major limitation of the concept of R0, which is an average and does not capture the heterogeneity of infectiousness [6]. Each infected case does not produce R0 offspring; a small number of individuals may be responsible for a large percentage of secondary infections, whereas most others infect no one. When this occurs, the offspring distribution is said to be over-dispersed. The dispersion parameter, k is smaller when superspreading plays a larger role in transmission. Studying overdispersion is crucial since most of the transmission can be eliminated if events and settings conducive to superspreading can be limited by implementing targeted measures, as opposed to overarching policies that would be needed if overdispersion was low and transmission was homogeneous [7, 8].

Serial interval, defined as the interval between symptom onset of the index case and the secondary case in a transmission chain, is a key epidemiological measure that determines the spread of an infectious disease. The serial interval is an essential metric in epidemic transmission models and in estimating reproduction numbers used to evaluate the impact of interventions and to inform policy response [9]. Studies across the world estimate the serial interval of SARS-CoV-2 between 3.9–7.5 days [10]. Estimation requires high quality data from primary contact tracing which establishes linkage between transmission pairs and thus, data-backed evidence of serial interval of SARS-CoV-2 in India and other low-resource settings is extremely limited if any. Whereas the secondary attack rate which measures the risk of infection in contacts has been studied in India by Laxminarayan et al. [11], a comprehensive study looking at superspreading, overdispersion and serial interval has not yet been done in India, mostly owing to a lack of data.

Karnataka has had the third-highest COVID-19 case burden of all states in India. Contact tracing of COVID-19 in Karnataka was driven by multi-sectoral teams and backed by technology, enabling the state to have one of India’s most effective contact tracing systems, at least during the early epidemic [12]. Among all Indian states, Karnataka was found to have the highest quality of COVID-19 data reporting in daily bulletins released by the state government [13]. In this study, we aimed to gain insights for disease control by understanding the transmission dynamics of SARS-CoV-2 based on surveillance and tracing data from Karnataka. We estimated the reproduction number (R), overdispersion (k), secondary attack rate (SAR), and serial interval from data and reconstructed major transmission networks. We evaluated the effect of age and other factors on the probability of asymptomatic infection, risk of transmitting the infection further, and mortality due to COVID-19. We also attempt to quantify the effect of contact tracing on reducing onward transmission and on minimising testing delays.

## Methods

### Data sources

We used data generated through surveillance activities undertaken by the Integrated Disease Surveillance Program (IDSP) and the Department of Health and Family Welfare in accordance with national and state policies. Data was de-identified before extraction and analysis. We used two separate datasets and merged them using the unique patient ID that was common to both datasets. The first dataset was sourced from daily COVID-19 bulletins released by the Government of Karnataka [14] (n = 71068 cases; 9 March to 21 July 2020, after which individual case details were not available in daily bulletins). Variables in this dataset included—age, sex, district of reporting, case outcome (followed up till 23 August), the unique ID of upstream contact (if known), travel history (if any), and case surveillance definition (if assigned). The second dataset comprised of linelist contact tracing data maintained by the IDSP (n = 3404 cases; 9 March to 1 June 2020) [15, 16]. This linelist data added the following variables—symptom status (asymptomatic or symptomatic) at time of sample collection, date of symptom onset (n = 261/308 symptomatic cases), date of sample collection and test results, and number of contacts traced (n = 956/3404 cases). As such, analyses that required data from the IDSP dataset (estimation of secondary attack rate, determinants of risk of infection in contacts, determinants of symptomatic infection in cases, and serial interval) were limited to the timeframe of the second dataset (9 March to 1 June 2020). Non-pharmaceutical interventions in effect during the study period included mandated physical distancing and mask-wearing in public spaces, closures of schools, restricted access to public places, and bans on large gatherings. The study followed the Strengthening the Reporting of Observational Studies in Epidemiology (STROBE) reporting guidelines [17].

### Case identification and contact tracing

The criteria for administering a COVID-19 test were periodically updated by the Indian Council of Medical Research (Table S1 in S1 File) [18]. A contact was defined as any individual who has been exposed to a confirmed case anytime between 2 days prior to symptom onset (in the positive case) and date of isolation (or maximum 14 days after onset of symptoms). All contacts were quarantined and monitored for 14 days. The duration (>15 minutes), the proximity (<1 meter), and the nature of exposure were taken into consideration when defining a contact [19]. Contacts were further divided into high-risk or low-risk contacts (S1 Section in S1 File). A COVID-19 test was administered to all symptomatic contacts and high-risk asymptomatic contacts between day 5 to 10 after contact [20]. For interstate and international travellers, screening was done at all ports of entry including airports, train stations and road checkposts at state borders. Symptomatic travellers were tested and asymptomatic travellers were subject to 14 days of quarantine [21, 22].

### Case categorisation and ascertainment of transmission pairs

We divided all cases into four mutually exclusive categories based on origin of infection: imported international (history of international travel within 14 days of symptom onset), imported domestic (history of travel from other Indian states into Karnataka), local with known origin (contact with a known COVID-19 case or present at a location or event linked to COVID-19 transmission, and no travel history outside Karnataka), and local cases with unknown origin (no travel history outside Karnataka and could not be traced to any known COVID-19 case or event). Additionally, we categorised cases on the basis of their surveillance case definition at the time of first presentation: Influenza-like illness (ILI: fever ≥38 C° and cough with onset within the last ten days), or Severe acute respiratory infection (SARI: fever ≥ 38C° and cough with onset within the last 10 days and requires hospitalization). This categorisation was independent of the categories based on origin and was assigned for only cases for whom this information was available. If a newly confirmed case had a history of contact with a previously confirmed case, we assumed that the new case acquired the infection from the previously known case; and hence linked them together as secondary case and index case respectively (probable infector-infectee pair). If a secondary case was in contact with 2 or more index cases, we linked the secondary case to the earliest index case.

### Reproduction number (R) and overdispersion parameter (k)

We fit a negative binomial distribution to the observed offspring distribution using a Bayesian Markov Chain Monte Carlo approach and estimated effective reproduction number (R) as the mean, and degree of heterogeneity in transmission as the overdispersion parameter (k) of the fitted distribution. Sample mean and 2.5% and 97.5% percentiles based on post warmup samples were used to evaluate the posterior point estimates and 95% confidence intervals respectively. Since estimates of R and k are sensitive to the completeness of contact tracing, we separately analysed subgroups where contact tracing was known or expected to be comprehensive (S5 Section and Table S2 in S1 File). Specific metrics of contact tracing adequacy and performance were not available in the dataset [23]. A cluster was defined as two or more confirmed infections with reported close contact. The superspreading threshold was defined as an index case causing eight or more secondary infections [24].

### Secondary attack rate, determinants of risk of infection among contacts and risk of symptomatic infection among cases, and case fatality rate

We computed secondary attack rates by dividing the number of positive contacts by the number of contacts traced. Only high-risk or close contacts were included (S1 Section in S1 File). We did a retrospective cohort analysis using a Poisson regression model to estimate adjusted relative risks of infection in contacts, and symptomatic infection in cases as a function of the predictor variables (S3 and S4 Sections in S1 File) [25]. Case fatality rate was calculated as the number of deceased cases divided by total reported cases.

### Serial interval

The serial interval was calculated as the difference between the symptom onset dates of index case and secondary case in each transmission pair. Only high-risk or close contacts were included in determining these transmission pairs (S1 Section in S1 File). We then fitted parametric distributions (gamma, lognormal, and weibull) to serial interval data using the maximum likelihood method. Akaike Information Criterion (AIC) was used to choose the optimal distribution, and confidence intervals were estimated using 10000 bootstraps.

### Data handling

Data collection was done in Microsoft Office Excel. Data cleaning and analysis was done in Stata 15.0 and Python v3.6.8. Figures were prepared using matplotlib v3.3.2, seaborn 0.10.0, and GraphPad Prism 9. Clusters were visualised using *EpiContacts* package in R [26].

### Ethics approval

Surveillance and contact tracing activities wherein the data was collected were part of ongoing outbreak investigation mandated by state and national health authorities. The requirement for full review was waived by the Institutional Ethics Committee, Indian Institute of Public Health, Bengaluru of Public Health Foundation of India (IIPHHB/TRCIEC/211/2020 dated 25/12/2020).

## Results

**Fig 1:**
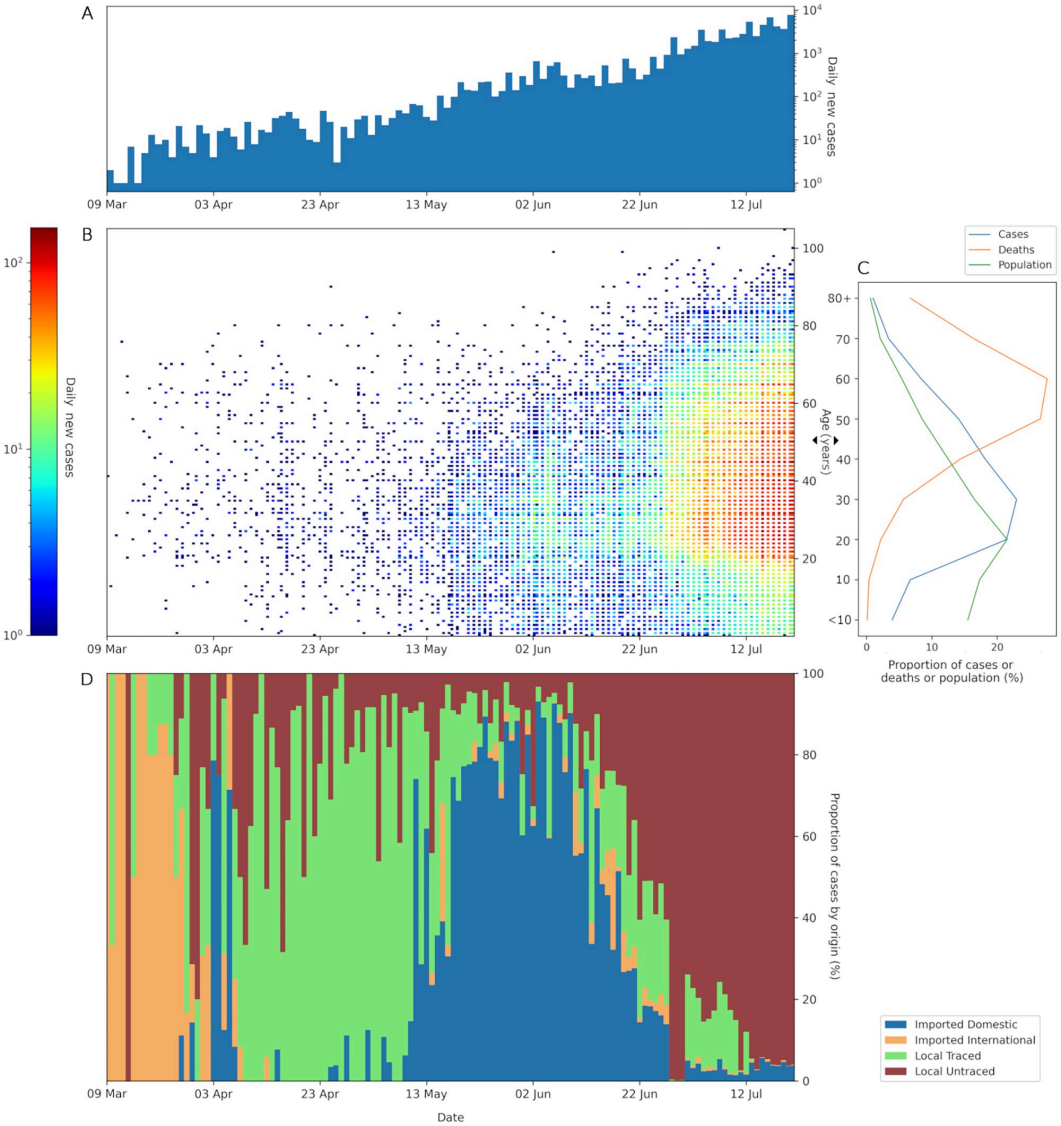
Epidemiological summary of the COVID-19 outbreak in Karnataka, India from 9 March to 21 July 2020 (n = 71068). **[A]** Number of daily confirmed cases across time. **[B]** Density scatterplot showing daily confirmed cases for various ages across time. **[C]** Distribution of population, COVID-19 cases and COVID-19 deaths across age groups. 10-year age brackets have been used starting from 0–9 years, and the last bracket is ≥80 years. **[D]** Daily confirmed cases across time stratified by origin of infection- imported international, imported domestic, local with known origin, and local with unknown origin.

From 9 March to 21 July 2020, Karnataka reported 71068 cases with a median age of 37 years (IQR 27–50) of which 63.1% were males. Table 1 and Fig 1 give an epidemiologic summary of the studied outbreak with time and demographic characteristics. Early in the epidemic, most cases occurred in middle-aged adults which can be explained by their high mobility. A ‘V’ shaped pattern can be seen in Fig 1C, indicating that more older adults and children were infected with time. Daily new cases increased throughout the study period, with 55826 (78.55%) cases recorded in July alone. Most cases in March were internationally imported followed by local transmission in April and May. A majority of the 5105 local cases whose origin was known occurred from April to mid-June. The source of infection of 57670 (81.15%) locally transmitted cases could not be ascertained; most of these cases occurred after mid-June. 20.3% of all cases were detected through symptomatic surveillance (17.1% ILI and 3.2% SARI). The median age of ILI cases was 40 (IQR 29–53) and SARI cases was 54 years (IQR 43–65). The mean delay from symptom onset to lab confirmation (testing delay) was 5.1 (4.7–5.6) days (n = 261). Local cases with unknown origin had the longest delay among all categories at 5.7 (5.1–6.2) days (n = 112). Detailed results of various delays are given in Table S4 in S1 File.

**Table 1:** Distribution of 71068 COVID-19 cases across time and categories and their demographic characteristics and case fatality rate (Karnataka, India; up to 21 July 2020).

	Number of cases, n (%)	Median age (IQR)	Gender Male, %	Case Fatality Rate, % (95% CI)
**Month**				
March	101 (0.14%)	35 (25–50)	68.32	1.98 (0.2–7.0)
April	464 (0.65%)	35 (25–48)	67.67	5.39 (3.5–7.8)
May	2656 (3.74%)	29 (19–39)	59.94	1.13 (0.8–1.6)
June	12021 (16.91%)	34 (24–48)	62.37	3.18 (2.9–3.5)
July	55826 (78.55%)	38 (28–52)	63.35	2.47 (2.3–2.6)
**Category (origin of infection)**				
Imported Domestic	7640 (10.75%)	30 (20–40)	63.31	0.46 (0.3–0.6)
Imported International	653 (0.92%)	35 (25–42)	76.26	0.15 (0.0–0.8)
Local with known origin	5105 (7.18%)	32 (22–45)	55.81	0.84 (0.6–1.1)
Local with unknown origin	57670 (81.15%)	39 (28–52)	63.56	3.02 (2.9–3.2)
**Category (surveillance case definition)**				
ILI	12124 (17.06%)	40 (29–53)	68.99	2.42 (2.2–2.7)
SARI	2295 (3.23%)	54 (43–65)	66.54	23.66 (21.9–25.4)
**Total**	71068	37 (27–50)	63.09	2.54 (2.4–2.7)

For case fatality rate, month is according to date of case confirmation. ILI = Influenza like illness, SARI = Severe acute respiratory infection.

### Overdispersion (k), reproduction number (R), and major transmission clusters

**Fig 2:**
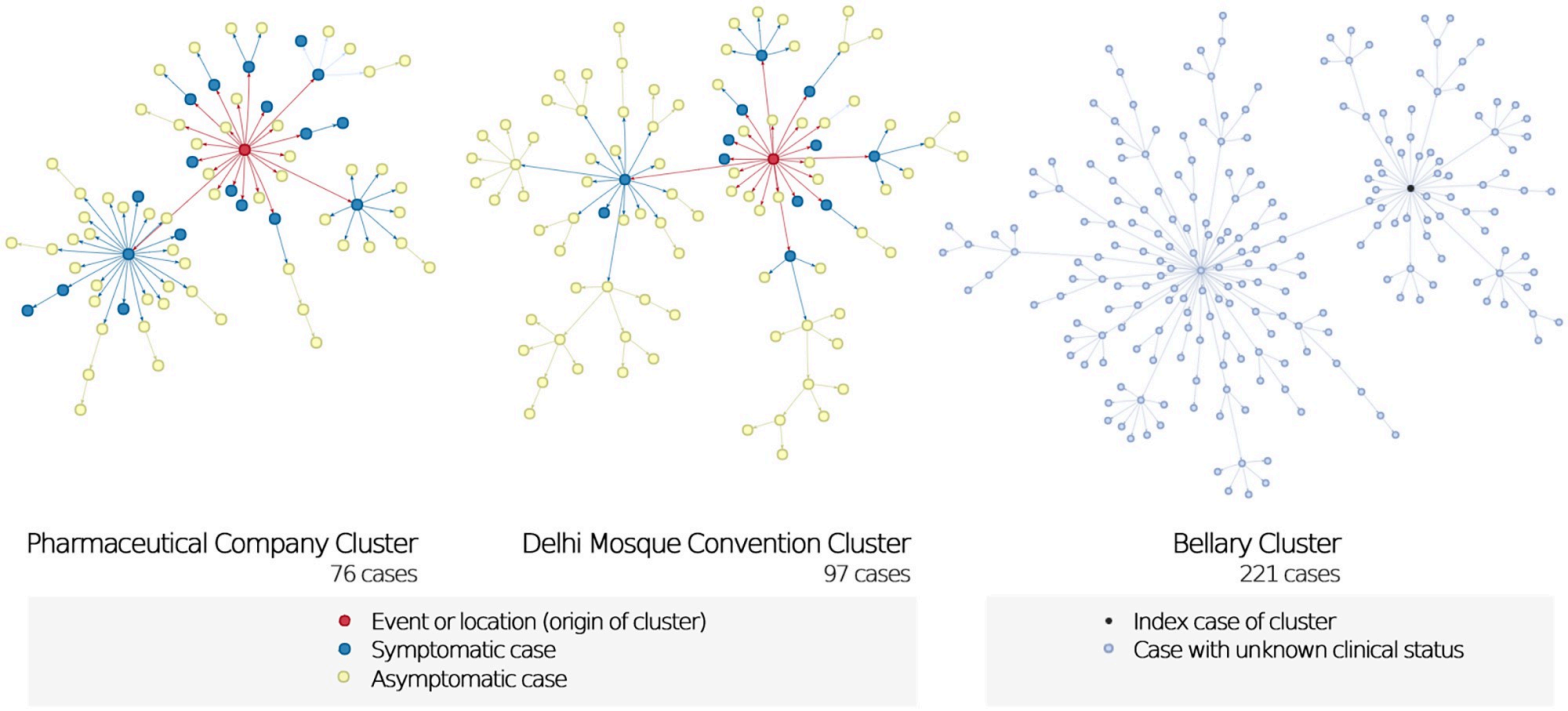
Transmission networks of three largest clusters of SARS-CoV-2 cases in Karnataka, India up to 21 July 2020. The networks indicate the heterogeneity in transmission from infected cases, with a few patients causing most secondary cases. The Bellary cluster occurred in June and July, during which symptomatic status of cases was not available.

**Fig 3:**
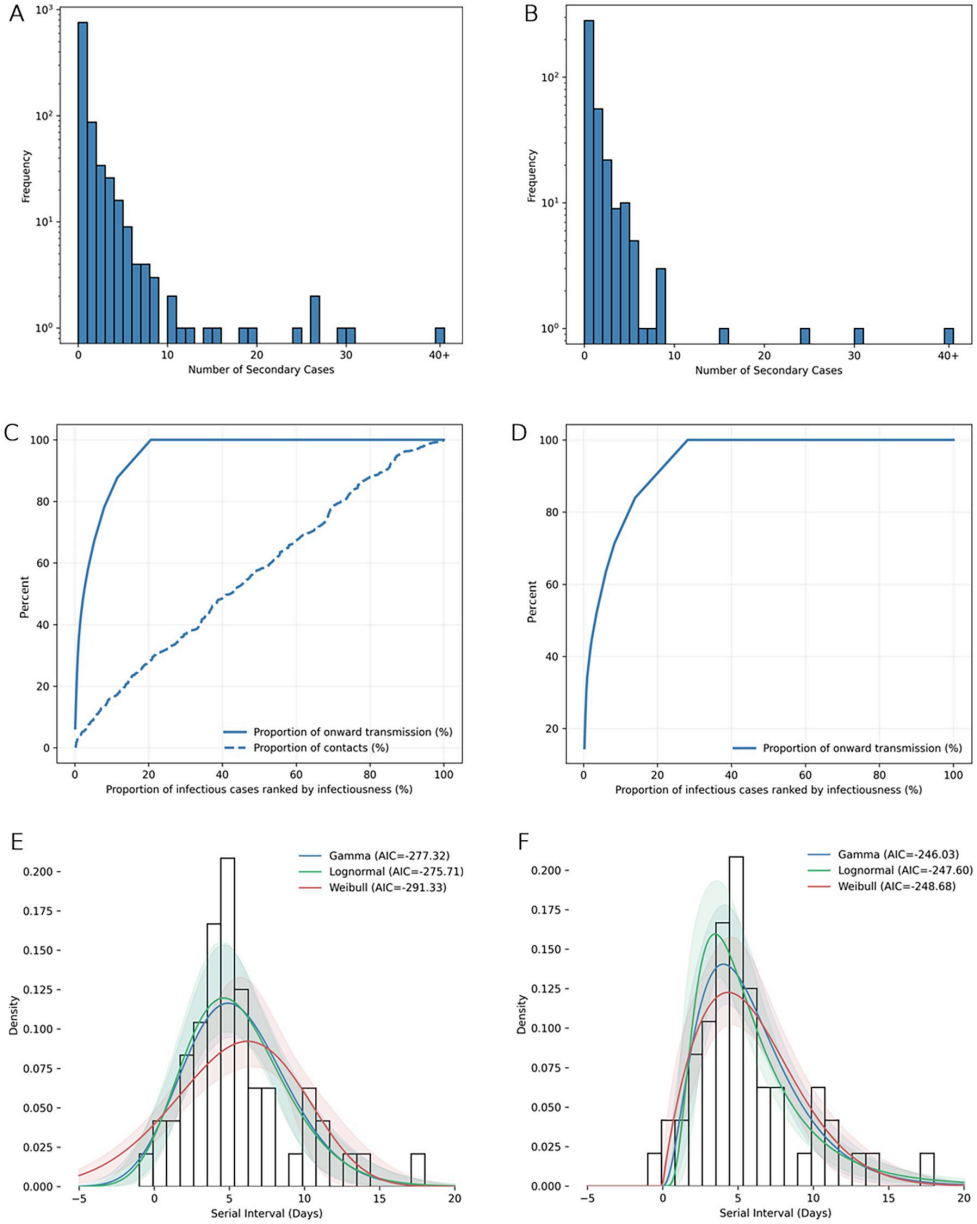
Transmission characteristics of SARS-CoV-2 in Karnataka, India. **[A, B]** Observed offspring distribution of [A] 956 cases with confirmed forward contact tracing and [B] 394 cases linked to the three largest clusters (Bellary cluster, Delhi convention cluster, and Pharmaceutical company cluster). Bars show observed frequency of the number of individuals infected by each case. **[C, D]** Proportion of all onward transmission (solid line) and proportion of all contacts (dashed line) due to a given proportion of infectious cases, where infectious cases are ranked by number of offspring cases (ie, infectiousness), for [C] 956 cases with confirmed forward contact tracing and [D] 394 cases linked to the three largest clusters. **[E, F]** Serial interval distribution of SARS-CoV-2 infections in Karnataka, India and the fitted gamma, lognormal and weibull distributions, among [E] 53 infector-infectee pairs and [F] 51 infector-infectee pairs with positive serial intervals. 10000 bootstraps were used for estimating the 95% confidence limits shown by the shaded bands.

We found 111 instances where the index case crossed the super-spreading threshold of ≥8 secondary cases upto 21 July 2020 in Karnataka. Among 111 super-spreader index cases, 50.5% were aged 20–39 years, 77.5% did not have a known infectious source, none were internationally imported, and median (IQR) number of secondary cases of each index were 12 (9–15). A total of 1277 clusters were identified with 6424 linked cases till 21 July 2020 in Karnataka. There were 7 clusters with a size larger than 50 cases and 106 clusters with more than 10 cases each. We characterised the three largest clusters including the Bellary cluster (221 cases), the Delhi convention cluster (97 cases), and the Pharmaceutical company cluster (76 cases). The reconstructed transmission networks for these three clusters are shown in Fig 2. For 394 cases linked to these three largest clusters, the estimated R was 0.91 (0.72–1.15) and k was 0.22 (0.17–0.27). 12.4% of infectious cases caused 80% of all transmission, while 71.2% of cases did not lead to any further transmission among these three clusters (Fig 3D). In late-March, a cluster of infections was seeded by travellers returning from the religious convention at Nizammudin mosque in Delhi. In the Bellary cluster, the infection was seeded by a worker at a steel plant in Bellary [27], followed by rapid spread among the employees living in close-contact in dormitories. The initial infections occurred in the management department which had a high contact rate with employees from other departments thus facilitating spread. A similar cluster was reported in Nanjangud [28], Mysore where a pharmaceutical company became the origin of a large cluster.

For 956 cases whose contacts were confirmed to have been traced, the estimated R was 0.75 (0.62–0.91) and k was 0.12 (0.11–0.15). 8.7% of infectious cases had 14.4% of contacts but caused 80% of all transmission (Fig 3C). On the other hand, 79.4% of cases did not lead to any further transmission despite having 71.6% of all recorded contacts. Asymptomatic cases had a much lower R than symptomatic cases (0.41 [0.32–0.52] vs 2.04 [1.56–2.67]), but had higher transmission heterogeneity indicated by a lower k (0.12 [0.09–0.15] vs 0.29 [0.23–0.37]). For all cases till 13 June (n = 6824), R was 0.23 (0.20–0.26) and k was 0.04 (0.03–0.04). Complete results for R and k are given in S5 Section and Table S2 in S1 File.

### Secondary attack rate and determinants of risk of infection among contacts

The secondary attack rate (SAR) among all close contacts was 3.6% (95%CI, 3.4–3.9). Results are summarised in Tables 2 and 3. The median number of contacts identified for an index case were 11 (IQR 5–21). The median number of contacts per case were higher (15 [IQR 8–35]) when the index case was confirmed ≥4 days after symptom onset. Symptomatic index cases were more infectious than asymptomatic cases (SAR 7.7% vs 2.0%; aRR 3.63 [3.04–4.34]). As compared to infectors aged 19–44 years, children were less infectious even after controlling for other factors including symptomatic status (aRR = 0.21 [0.07–0.66] for 0–5 years and 0.47 [0.32–0.68] for 6–18 years). Adults aged ≥45 years seemed to be more infectious, but this association disappeared when controlling for increased symptomaticity of older adults. Male infectors were found to be less infectious than females (aRR 0.78 [0.66–0.92]). Infectors who had a delay from symptom onset to confirmation of ≥4 days were associated with higher infectiousness (aRR = 3.01 [2.11–4.31]). Among the four categories based on epidemiological origin of infection, local cases with unknown origin had the highest infectivity with R of 1.04 (0.87–1.25) and SAR of 8.5% (7.7–9.4). Cases identified through symptomatic surveillance had a high SAR (14% and 9.8% for ILI and SARI respectively).

**Table 2:** Secondary attack rate of SARS-CoV-2 among 16715 contacts of 956 index cases (Karnataka, India; up to 1 June 2020).

	Median number of contacts per index case, n (IQR)	Positive contacts/ Total contacts	SAR, % (95%CI)
**Month**			
March	10.5 (6–21.25)	59/1408	4.2 (3.2–5.4)
April	12 (6–27)	349/7120	4.9 (4.4–5.4)
May	10 (5–28)	202/8187	2.5 (2.1–2.8)
**Symptom status of index case**			
Symptomatic	13 (7–28)	367/4788	7.7 (6.9–8.4)
Asymptomatic	10 (5–20)	243/11927	2.0 (1.8–2.3)
**Category (origin of infection) of index case**			
Imported international	10 (5.25–18.5)	24/1075	2.2 (1.4–3.3)
Imported domestic	9 (3–20)	47/4670	1.0 (0.7–1.3)
Local with known origin	11 (6–20)	178/6733	2.6 (2.3–3.0)
Local with unknown origin	12 (7–13)	361/4237	8.5 (7.7–9.4)
**Category (surveillance case definition) of index case**			
ILI (not requiring hospitalisation)	17 (9–31)	87/622	14.0 (11.4–17.0)
SARI (requiring hospitalisation)	16 (10–43)	134/1371	9.8 (8.3–11.5)
**Delay from symptom onset to confirmation of index case**			
<4 days	10.5 (6–20.25)	41/1091	3.8 (2.7–5.1)
≥4 days	15 (8–35)	313/3256	9.6 (8.6–10.7)
**Overall**	**11 (5–21)**	**610/16715**	**3.6 (3.4–3.9)**

**Table 3:** Risk of SARS-CoV-2 infection among 16715 contacts of 956 index cases (Karnataka, India; up to 1 June 2020).

	Crude RR (95%CI)	Adjusted RR* (95%CI)
**Age group of index case**		
0–5 years	0.22 (0.07–0.69)	0.21 (0.07–0.66)
6–18 years	0.40 (0.27–0.59)	0.47 (0.32–0.68)
19–44 years	1 (reference)	1 (reference)
45–64 years	1.47 (1.22–1.77)	1.02 (0.85–1.23)
≥65 years	1.45 (1.16–1.82)	0.89 (0.70–1.13)
**Sex of index case**		
Female	1 (reference)	1 (reference)
Male	0.89 (0.76–1.05)	0.78 (0.66–0.92)
**Symptom status of index case**		
Asymptomatic	1 (reference)	1 (reference)
Symptomatic	3.76 (3.21–4.41)	3.63 (3.04–4.34)
**Delay from symptom onset to lab confirmation in index case** **		
<4 days	1 (reference)	1 (reference)
≥4 days	2.56 (1.86–3.52)	3.01 (2.11–4.31)

* Adjusted for age group, sex and symptom status of index case.

** Adjusted for age group and sex of symptomatic index case. This predictor variable was part of a separate model that included only symptomatic index cases and their contacts.

### Serial interval

After excluding asymptomatic cases, 54 infector-infectee pairs were identified where symptom onset dates for both infector and infectee were available. 35, 14 and 5 pairs were from March, April and May respectively. One pair was dropped for having a large negative serial interval (-19 days). Estimated parameters for the serial interval distribution are shown in Table S3 in S1 File and the fit is shown in Fig 3E, 3F and S3 Section in S1 File. When analysing the 53 pairs, Weibull distribution was the best fit (AIC = -291) with a mean of 5.4 (4.4–6.4) and SD of 4.3 (3.0–5.1) days. When analysing serial intervals with positive values only (51 pairs), the Weibull distribution was again the best fit (AIC = -249) with a mean of 5.9 (5.0–6.9) and SD of 3.3 (2.4–4.1) days.

### Determinants of presence of clinical symptoms in SARS-CoV-2 infection

Among 3404 cases till 1 June, 9.0% were symptomatic at the time of sample collection. Increasing age, male sex, and having a symptomatic infector were associated with a higher probability of being symptomatic given SARS-CoV-2 infection (Table 4). The probability of symptomatic infection increased with age. When compared to cases aged 19–44 years old, children were about half as likely to be symptomatic (aRR = 0.46 [0.19–1.11] for 0–5 years and 0.39 [0.24–0.64] for 6–18 years); whereas older adults aged 45–64 years were about twice as likely to be symptomatic (aRR = 2.24 [1.77–2.84]), and elderly aged ≥65 years were about four times as likely to be symptomatic (aRR = 4.46 [3.37–5.90]). Males were 1.29 (1.04–1.62) times more likely to be symptomatic than females. A secondary case was 8.16 (3.29–20.24) times more likely to be symptomatic if the index case was also symptomatic compared to if it was asymptomatic. Presence of one or more comorbidities was associated with increased symptomaticity but was not statistically significant (aRR = 1.13 [0.92–1.39]).

**Table 4:** Symptomatic proportion and risk of developing symptoms among 3404 COVID-19 cases (Karnataka, India; up to 1 June 2020).

	Symptomatic cases, n (%)	Crude RR (95%CI)	Adjusted RR* (95%CI)
**Age group of case**			
0–5 years (n—147)	5 (3.4)	0.46 (0.19–1.09)	0.46 (0.19–1.11)
6–18 years (n—602)	17 (2.8)	0.38 (0.23–0.62)	0.39 (0.24–0.64)
19–44 years (n—1955)	146 (7.4)	1 (reference)	1 (reference)
45–64 years (n—570)	96 (16.8)	2.26 (1.77–2.87)	2.24 (1.77–2.84)
≥65 years (n—130)	44 (33.8)	4.53 (3.40–6.03)	4.46 (3.37–5.90)
**Sex of case**			
Female (n—1309)	97 (7.4)	1 (reference)	1 (reference)
Male (n—2095)	211 (10.1)	1.36 (1.08–1.71)	1.29 (1.04–1.62)
**Comorbidity in case**			
Present (n—1043)	117 (11.2)	1.39 (1.11–1.72)	1.13 (0.92–1.39)
Absent (n—2361)	191 (8.1)	1 (reference)	1 (reference)
**Symptom status of index case** **			
Asymptomatic (n—370)	5 (2.9)	1 (reference)	1 (reference)
Symptomatic (n—455)	55 (12.1)	8.95 (3.62–22.11)	8.16 (3.29–20.24)
**Overall (n—3404)**	307 (9.0)	-	-

*Adjusted for age group, sex and presence of comorbidity in affected case, and symptom status of the index case.

**Symptom status of index case was not available for 2579 out of 3404 cases whose infector was unknown (these cases were included in the model by making a separate category not shown in the table).

### Case fatality rate

**Fig 4:**
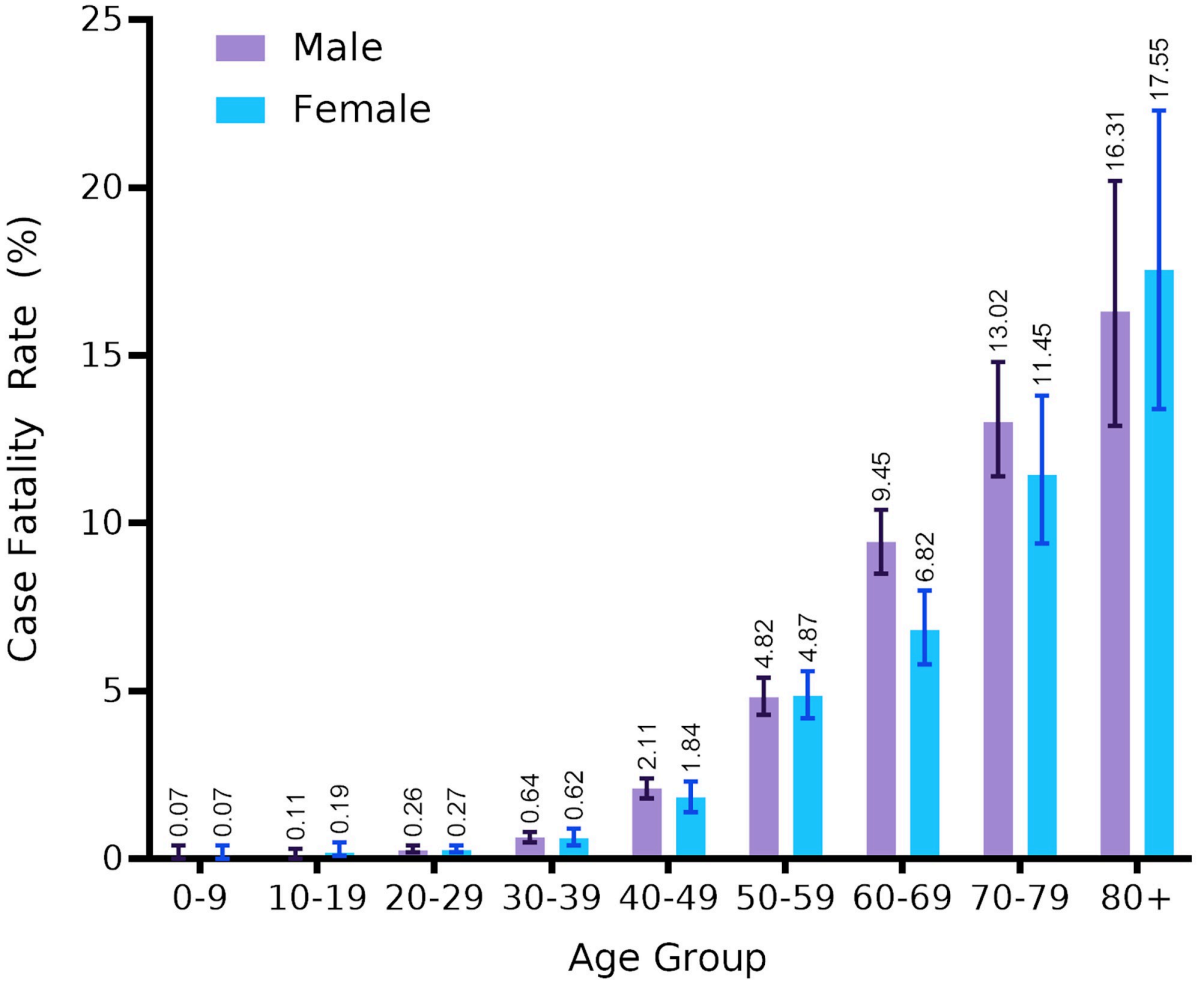
Case fatality rate COVID-19 cases stratified by age and sex in Karnataka, India up to 21 July 2020 (n-71068). Bars represent mean CFR for that subgroup (value shown above bars) with error bars showing the 95% confidence intervals.

The case fatality rate (CFR) across time and case categories is presented in Table 1 and across age and sex in Fig 4. Among 71068 cases, the overall CFR was 2.5% (95% CI, 2.4–2.7%). Local cases with unknown origin had the highest CFR (3.0%), followed by local cases with known origin (0.84%), imported domestic cases (0.46%), and imported international cases (0.15%). In surveillance case categories, SARI cases had a much higher CFR (23.66%) than ILI (2.42%). CFR steadily increased with age in both sexes, ranging from 0.07% in 0–9 years old to 16.31% in males and 17.55% in females of age 80 and above (Fig 4). The highest CFR was recorded for cases that were reported in the month of April (5.39%).

## Discussion

Although the reproduction number R0 of the novel coronavirus has been well characterised, we find that R0 alone fails to capture the true picture of individual-level transmission dynamics. Overdispersion (k) ranged from 0.04 to 0.34 in our study, confirming that there is significant heterogeneity in transmission of the novel coronavirus. Importantly, we did not find significant underlying heterogeneity in the number of contacts. Fig 3C shows clustering in the number of secondary cases (concave line) while the number of contacts are homogenous as indicated by a relatively linear relation. Heterogeneity in transmission can be explained by heterogeneity in the number of contacts and/or the probability of infection per contact (infectivity level of index case and nature of exposure) [29, 30]. Modelling studies indicate that SARS-CoV-2 SSEs occur when an infected person is briefly shedding at a very high viral load and has a high concurrent number of exposed contacts [31]. Since the patients which caused a majority of secondary cases did not have a concurrent larger share of total contacts (8.7% of infectious cases had 14.4% of contacts but caused 80% of transmission), our findings underscore the importance of the high infectivity of index case (at the time of exposure) and the nature of exposure in causing a successful SSE.

The most reliable estimates of overdispersion (k) in our study were 0.12 (0.11–0.15) for confirmed traced cases (n = 956) and 0.22 (0.17–0.27) from the three clusters (n = 394). Our estimates of k align with the lower range of current global estimates, albeit with smaller confidence intervals due to larger sample size. A modelling study analysing global clusters estimated k at 0.10 (0.05–0.20) [32]. A study of 1288 cases estimated k at 0.06 (0.05–0.07) and 0.20 (0.09–0.31) in two states of Indonesia [33]. In China, k was estimated at 0.25 (0.13–0.88) from 135 cases in Tianjin [34], and at 0·58 (0·35–1·18) from 391 cases in Shenzhen [35]. In Hong Kong, k was estimated at 0.43 (0.29–0.67) from 290 cases [24]. In Georgia USA, the overall k ranged from 0.32 to 0.49, with even lower values after shelter-in-place orders were issued [36]. Our findings suggest that super-spreading played a more dominant role in transmission in Karnataka, India, as compared to most high-income countries. Interestingly, phylogenetic studies indicate that remote clusters can be retrospectively linked to a previous SSE, indicating that super-spreading may play an even larger role in the overall propagation of the epidemic than is detected through surveillance and tracing [37]. Ideally then, the true picture of epidemics can only be understood by analysing contact tracing, serological and phylogenetic data together, which can help plan adequate control measures.

Modelling studies indicate that the delay from symptom onset to confirmation (testing delay) is an essential determinant of the effectiveness of contact tracing [5, 38]. Indeed, we found that infectors diagnosed four or more days after symptom onset led to a higher SAR among their contacts (9.6% vs 3.8%) and also had a higher number of contacts (median 15 vs 10.5). We found that this delay was lower for cases who had an early first contact with surveillance or contact tracing systems, namely, cases screened at entry ports (imported cases) and local cases from known contact lists. Imported cases had a low reproduction number compared to local cases (Table S2 in S1 File) which suggests that screening and registration of all persons at entry ports leading to early detection of these cases prevented most onward transmission. Cases captured through symptom-based surveillance and those whose source of infection was unknown had a higher SAR than all other case categories (Table 2) and had a concurrently higher testing delay (Table S4 in S1 File). SARI had lower infectiousness than ILI cases which could be explained by their hospitalisation preventing some transmission. Cases that were from known contact lists were confirmed 0.88 days (0.60–2.04) earlier and caused 60% less secondary infections than local cases whose origin was unknown (R 1.04 vs 0.42). These findings highlight the effect of contact tracing in reducing transmission and reassert the importance of minimising testing and tracing delays.

We estimated the relative risk (aRR) of infectivity at 3.63 (3.04–4.34) for symptomatic infectors as compared to asymptomatic carriers (SAR 7.7 vs 2.0%; R 2.04 vs 0.41) (Table 2 and S2 Section in S1 File). Other studies have found that infectiousness also increases with disease severity [39]. Accordingly, interventions aimed at children might have a relatively small impact on reducing SARS-CoV-2 transmission, as suggested by their low symptomaticity and severity and thus lower infectiousness than adults [40]. Our study also finds that children aged 6–18 are less infectious even after adjusting for lower symptomatic infections in this group. Interestingly, we found a strong association between the symptom status of the index and secondary case; symptomatic infectors were 8.16 (3.29–20.24) times more likely to generate symptomatic secondaries. These findings are corroborated by He D et al., who estimated relative risk of infectivity of symptomatics against that of asymptomatics at 3.9 (1.5–11.8), and at 6.6 (2.0–34.7) when focusing on symptomatic secondaries [41]. With symptomatic infectors both, more likely to produce secondary cases in general and also more likely to produce symptomatic secondaries who are themselves more infectious, it seems that a cascading effect of high transmission potential may play a role in amplifying COVID-19 outbreaks. Though it is known that nasopharyngeal viral load is higher in symptomatic cases and increases with severity in COVID-19 [42], it would be pertinent to explore whether a higher infectious dose from the infector influences symptom status and infection severity in the secondary case, something that could explain findings from this work and He D et al. [41, 43].

We found one study estimating the serial interval of SARS-CoV-2 from contact tracing data in India; however, this study assumed the date of sample collection in asymptomatic cases as a proxy for symptom onset which heavily affects the reliability of their estimate [44]. Although we present reliable estimates of serial interval for COVID-19 for the first time from India, enhanced data sharing enabling real-time estimation to inform policy decisions is recommended to account for the temporal variation of serial interval as observed in China [45]. Given that the reproduction number is sensitive to the value of serial interval used for its estimation, it is prudent to select the serial interval distribution that fits best in context of the location and time phase of the epidemic [46]. Our estimated mean serial interval of 5.4 (4.4–6.4) agrees with existing evidence from global studies [10].

Our study has certain limitations. Firstly, symptomatic status was based on data collected at the time of sample collection and hence some cases recorded as asymptomatic may have developed symptoms later [47–49]. This would overestimate the proportion of asymptomatic infections and also the relative transmissibility by asymptomatics since presymptomatic cases have been shown to be more infectious than asymptomatic carriers [41, 48, 50]. Second, any amount of case and/or contact under-ascertainment during surveillance and contact tracing carries the potential to bias our results. Although we have attempted to minimise this bias by analysing subgroups with high reliability of data (Table S2 in S1 File), some degree of bias can still be expected. Third, details of settings of transmission and timing of exposure of contact to index case were not available for the vast majority of cases which precluded any insightful analysis on the same. Finally, the dates of symptom onset in our study may be subject to recall bias.

Our findings have a few important implications for optimizing policy. We find that even though surveillance, tracing, and social distancing may keep the reproduction number and hence transmission at low levels, super spreading is common in COVID-19 and carries the potential to acutely overwhelm surveillance and tracing systems. However, this presents an opportunity as well, in that outbreaks where a minority of cases cause most further transmission (high dispersion, low k) are much more amenable to control through measures that target the high-risk groups and settings responsible for most of the transmission. Though during our study duration the ancestral variant was the predominantly circulating variant, the more recently emerged Delta variant (B.1.617) and Omicron variant (B1.1.529) have been associated with increased transmissibility and waning vaccine effectiveness as compared to the ancestral strain. These observations suggest that greater emphasis should be placed on non-pharmacological interventions aimed at preventing super-spreading events to reduce the overall burden of COVID-19 [51–53]. Existing measures that limit potential super-spreading including bans on large gatherings and capping capacity in closed spaces are expected to remain beneficial [29]. Specifically, emphasis should be given on backward or retrospective contact tracing which becomes increasingly effective as overdispersion increases, and tracing and testing delays should be minimized [32, 38]. Evidence on the effect of symptom status on transmission suggests that measures targeted at children will not reduce transmission significantly [40, 50]. Although more studies are needed, there is increasing evidence that symptomatic cases beget more symptomatic secondaries and may cause a snowballing effect on transmission across generations [41], which has significant implications for both- transmission and morbidity control in COVID-19 outbreaks.

## Supporting information

S1 File (DOCX)
