# Correction: Contact tracing of COVID-19 in Karnataka, India: Superspreading and determinants of infectiousness and symptomatic infection

**DOI:** 10.1371/journal.pone.0298090

**Published:** 2024-01-26

**Authors:** Mohak Gupta, Giridara G. Parameswaran, Manraj S. Sra, Rishika Mohanta, Devarsh Patel, Amulya Gupta, Bhavik Bansal, Vardhmaan Jain, Archisman Mazumder, Mehak Arora, Nishant Aggarwal, Tarun Bhatnagar, Jawaid Akhtar, Pankaj Pandey, Vasanthapuram Ravi, Giridhara R Babu

The affiliation for the corresponding author is incorrect. The correct affiliation is not indicated. Giridhara R. Babu is not affiliated with #9 but with: Department of Population Medicine, College of Medicine, QU Health, Qatar University, Doha, Qatar.
