# Supplementary material for: Contact tracing of COVID-19 in Karnataka, India: Superspreading and determinants of infectiousness and symptomatic infection
Source: PLoS One. 2022 Jul 11;17(7):e0270789. doi: 10.1371/journal.pone.0270789 (PMC9273085; doi:10.1371/journal.pone.0270789)
Supplement: S1 File — (DOCX) [file pone.0270789.s001.docx]

**Supporting Materials**

**S1. Definition of high risk and low risk contact**^1^

High-risk contact:

- Lives in the same household as the case
- Anyone in close proximity (within 1 meter) of the confirmed case without precautions
- Touched or cleaned the linens, clothes, or dishes of the patient.
- Had direct physical contact with the body of the patient including physical examination without PPE.
- Passenger in close proximity (within 1 meter) of a conveyance with a symptomatic person who later tested positive for COVID-19.
- Touched body fluids of the case without appropriate PPE (respiratory tract secretions, blood, vomit, saliva, urine, feces)

Low-risk contact:

- Any contact not fitting into the above high-risk contact description.

**S2. Bayesian Markov Chain Monte Carlo sampling for estimating reproduction number and overdispersion from contact tracing data**

We implemented a Bayesian Markov Chain Monte Carlo sampling method using the NUTS sampler in PyStan 2.18.0.0 with gamma prior estimates of R and k with mean 2.5 SD 2.0, and mean 0.45 SD 0.1 respectively based on previous studies. ^2–5^ 8 chains were parallelly simulated for 5000 iterations and were checked for convergence of Rhat to 1. The first 500 iterations for all chains were discarded as a burn-in period.

**S3. Determinants of risk of infection among contacts**

We fit a Poisson regression model with robust error variance to data on contacts’ recorded infection status (binary outcome: contact infected OR not infected) and attributes of each contact's index cases to estimate adjusted relative risks for infection of contacts as a function of these exposures.^6^ For a subset of this data that included only symptomatic index cases and their contacts, we fit another model which also included the delay from symptom onset to confirmation of the index case (a proxy for delay in case isolation and increasing infectious period spent in community) as a predictor variable. Only high-risk/close contacts were included because the recording of ‘low-risk contact’ status was not deemed reliable in the accessed dataset.

**S4. Determinants of presence of clinical symptoms in SARS-CoV-2 infection**

We fit a Poisson regression model with robust error variance to data on confirmed COVID-19 cases’ symptom status (binary outcome: case is symptomatic OR asymptomatic) and attributes of each case and their index case (where known) to estimate adjusted relative risks for developing symptoms in a COVID-19 case as a function of these exposures.^6^

**S5. Minimizing bias due to incomplete contact tracing**

The accuracy of reproduction number (R) and overdispersion (k) estimates from data depends on the completeness and adequacy of the contact tracing efforts during the sampled time. For example, cases with zero linked secondary cases in the dataset could either be due to no further transmission from the case after adequate tracing, or due to inadequate or absent contact tracing of the case leading to failure of identification of secondary cases. To account for the possible bias arising out of inadequate contact tracing specially at later stages of the study, we estimated R and k for multiple time periods, and for cases from each category and from major clusters separately.

Three cut-off dates are evaluated in table S2 and figure S2, with the following rationale.

- 1 June was the last date for which the detailed IDSP dataset was available (refer to “Data sources” under Methods section).
- As the epidemic progressed, contact tracing systems were stretched leading to inadequate identification or follow-up of contacts of some cases. We assumed that prevailing contact tracing was adequate if the origin of at least 80% of daily cases was known.^7,8^ This threshold was breached consistently (for more than seven days in a row) after 13 June. This became our second cut-off date for R and k analysis (to minimize bias due to inadequate tracing after this date).
- 21 July was the last date for which the state government bulletins were available with individual case details (refer to “Data sources” under Methods section).

**S6. Additional details on surveillance and tracing methods utilized by the state of Karnataka, India**

Karnataka utilised a technology backed multi-sectoral approach to contact tracing with collaboration between health department and law enforcement personnel to effectively trace and track cases. Innovations in quarantine enforcement included a Quarantine Watch mobile app and community based Mobile Squads to monitor contacts in quarantine. It was made mandatory for all returnees and travellers coming to Karnataka to register on a web portal, which enabled comprehensive screening at all entry points and adequate follow up after entry. Additionally, the state carried out a physical and phone-based survey which covered 15M out of 17M households in Karnataka to identify and protect high-risk populations including the elderly, pregnant females, persons with comorbidities, and persons with an ILI or SARI case profile.

**Table S1. Criteria for testing of COVID-19 in various versions of the recommended strategy by the Indian Council of Medical Research (ICMR)** ^9^

|  | **v1** | **v2** | **v3** | **v4** | **v5** |
| --- | --- | --- | --- | --- | --- |
|  | **9 March** | **17 March** | **20 March** | **9 April** | **18 May** |
| 1. All symptomatic (ILI symptoms) individuals with a history of international travel in the last 14 days. | • | • | • | • | • |
| 2. All symptomatic (ILI symptoms) contacts of laboratory confirmed cases. | • | • | • | • | • |
| 3. All symptomatic (ILI symptoms) health care workers / frontline workers involved in containment and mitigation of COVID19. |  | • | • | • | • |
| 4. All patients of Severe Acute Respiratory Infection (SARI). |  |  | • | • | • |
| 5. Asymptomatic direct and high-risk contacts of a confirmed case (to be tested once between day 5 and day 10 of coming into contact). |  |  | • | • | • |
| 6. All symptomatic ILI within hotspots/containment zones. |  |  |  | • | • |
| 7. All hospitalised patients who develop ILI symptoms. |  |  |  |  | • |
| 8. All symptomatic ILI among returnees and migrants within 7 days of illness. |  |  |  |  | • |
| 9. No emergency procedure (including deliveries) should be delayed for lack of test. However, sample can be sent for testing if indicated as above (1-8), simultaneously. |  |  |  |  | • |

ILI case is defined as one with acute respiratory infection with fever ≥ 38◦C AND cough.  SARI case is defined as one with acute respiratory infection with fever ≥ 38◦C AND cough AND requiring hospitalization. Direct and high-risk contacts include those who live in the same household with a confirmed case.

**Table S2. Posterior estimates of reproduction number (R) and overdispersion parameter (k)**

|  | **Till 1 June (n=3404)** | | **Till 13 June (n=6824)** | | **Till 21 July (n=71068)** | |
| --- | --- | --- | --- | --- | --- | --- |
|  | **Reproduction number, R (95% CI)** | **Overdispersion parameter, k (95% CI)** | **Reproduction number, R (95% CI)** | **Overdispersion parameter, k (95% CI)** | **Reproduction number, R (95% CI)** | **Overdispersion parameter, k (95% CI)** |
| Only cases with confirmed forward contact tracing* (n=956) | **0.75 (0.62-0.91)** | **0.12 (0.11-0.15)** | - | - | - | - |
| All cases | 0.32 (0.27-0.38) | 0.05 (0.04-0.06) | 0.23 (0.20-0.26) | 0.04 (0.03-0.04) | 0.19 (0.18-0.20) | 0.04 (0.03-0.04) |
| **Symptomatic status of index case*** | | | | | | |
| Asymptomatic (n=753) | **0.41 (0.32-0.52)** | **0.12 (0.09-0.15)** | - | - | - | - |
| Symptomatic (n=203) | **2.04 (1.56-2.67)** | **0.29 (0.23-0.37)** | - | - | - | - |
| **Category (case origin)** | | | | | | |
| Imported international | 0.24 (0.14-0.40) | 0.32 (0.19-0.51) | 0.14 (0.09-0.22) | 0.29 (0.17-0.46) | 0.06 (0.04-0.09) | 0.25 (0.14-0.42) |
| Imported domestic | 0.10 (0.07-0.14) | 0.02 (0.01-0.03) | 0.06 (0.04-0.07) | 0.02 (0.01-0.02) | 0.07 (0.06-0.08) | 0.02 (0.02-0.02) |
| Local with known origin | 0.38 (0.30-0.48) | 0.12 (0.09-0.15) | 0.42 (0.35-0.51) | 0.10 (0.08-0.13) | 0.26 (0.23-0.30) | 0.07 (0.06-0.08) |
| Local with unknown origin | 1.33 (1.06-1.70) | 0.21 (0.17-0.26) | 1.04 (0.87-1.25) | 0.17 (0.14-0.21) | 0.22 (0.20-0.24) | 0.04 (0.03-0.04) |
| **Clusters**** | | | | | | |
| Bellary cluster (n=221) | - | - | - | - | **1.04 (0.76-1.40)** | **0.23 (0.17-0.30)** |
| Delhi convention cluster (n=97) | - | - | - | - | **0.84 (0.54-1.25)** | **0.34 (0.22-0.48)** |
| Pharmaceutical company cluster (n=76) | - | - | - | - | **0.80 (0.48-1.29)** | **0.32 (0.20-0.46)** |
| All three clusters (n=394) | - | - | - | - | **0.91 (0.72-1.15)** | **0.22 (0.17-0.27)** |

**Estimates in bold are from subgroups where contact tracing was known or expected to be comprehensive, and thus bias to be minimal.**

* This data was available only for cases confirmed up to 1 June as part of the IDSP dataset.

** All cases linked to these three clusters were included irrespective of any last date of confirmation. Cases and contacts linked to known clusters are more likely to be followed-up and tested than sporadic cases. As such, estimates from these subgroups are expected to minimize bias when compared to those from the entire dataset.

**Table S3: Serial interval of SARS-CoV-2 estimated by fitting parametric distributions to data**

| **Distribution** | **Mean** | **Standard Deviation** | **Median** | **IQR** | **Goodness of fit (AIC)*** |
| --- | --- | --- | --- | --- | --- |
| **All values of serial interval (53 pairs)** | | | | | |
| Gamma | 5.528 (4.585-6.528) | 3.475 (2.590-4.265) | 5.323 (4.412-6.296) | 4.661 (3.482-5.706) | -277.32 |
| Lognormal | 5.521 (4.578-6.525) | 3.466 (2.593-4.263) | 5.220 (4.332-6.176) | 4.579 (3.454-5.583) | -275.71 |
| Weibull | 5.377 (4.450-6.421) | 4.303 (3.050-5.112) | 5.622 (4.698-6.672) | 5.882 (4.080-7.052) | -291.33 |
| **Only positive values of serial interval (51 pairs)** | | | | | |
| Gamma | 5.880 (4.980-6.880) | 3.304 (2.437-4.150) | 5.274 (4.479-6.186) | 4.198 (3.152-5.190) | -246.03 |
| Lognormal | 5.967 (5.053-6.994) | 3.940 (2.719-5.302) | 4.980 (4.203-5.865) | 4.152 (3.129-5.126) | -247.6 |
| Weibull | 5.905 (4.992-6.917) | 3.328 (2.426-4.123) | 5.446 (4.624-6.409) | 4.561 (3.390-5.531) | -248.68 |

*If the difference in AIC > 10 between two fits on same data, the difference in fit is significant with lower AIC suggesting a better fit.

**Table S4: Estimates of various delays for COVID-19 cases up to 1 June 2020**

|  | **Sample size, n** | **Mean, days (95% CI)** | **SD, days (95% CI)** |
| --- | --- | --- | --- |
| **All cases** | | | |
| Symptom onset to sample collection | 261 | 3.038 (2.609-3.487) | 3.553 (2.757-4.387) |
| Symptom onset to lab confirmation | 261 | 5.146 (4.732-5.594) | 3.643 (2.864-4.471) |
| **Imported international** | | | |
| Arrival to symptom onset | 57 | 3.333 (1.771-5.404) | 7.022 (3.592-10.687) |
| Symptom onset to sample collection | 58 | 2.621 (1.828-3.569) | 3.393 (1.794-4.549) |
| Symptom onset to lab confirmation | 58 | 4.672 (3.897-5.603) | 3.339 (1.960-4.429) |
| **Imported domestic** | | | |
| Arrival to symptom onset | 28 | 1.929 (0.714-3.214) | 3.369 (2.223-4.235) |
| Symptom onset to sample collection | 40 | 2.675 (1.475-4.075) | 4.268 (2.549-5.735) |
| Symptom onset to lab confirmation | 40 | 4.850 (3.650-6.275) | 4.204 (2.495-5.638) |
| **Local with known origin** | | | |
| Symptom onset to sample collection | 51 | 2.667 (1.765-3.980) | 4.152 (1.481-6.628) |
| Symptom onset to lab confirmation | 51 | 4.784 (3.804-6.157) | 4.376 (1.741-6.943) |
| **Local with unknown origin** | | | |
| Symptom onset to sample collection | 112 | 3.554 (3.018-4.107) | 2.939 (2.402-3.493) |
| Symptom onset to lab confirmation | 112 | 5.661 (5.098-6.241) | 3.107 (2.557-3.599) |
| **Cases detected through symptom-based surveillance (ILI or SARI)** | | | |
| Symptom onset to sample collection | 75 | 3.480 (2.853-4.173) | 2.918 (2.121-3.689) |
| Symptom onset to lab confirmation | 75 | 5.560 (4.867-6.293) | 3.159 (2.410-3.827) |

**Supporting Material Figure Captions:**

**Figure S1: Histograms showing distributions of various delays for 261 symptomatic COVID-19 cases till 1 June 2020. [A]** Delay from symptom onset to sample collection. **[B]** Delay from symptom onset to lab confirmation**.** See estimates in table S4.


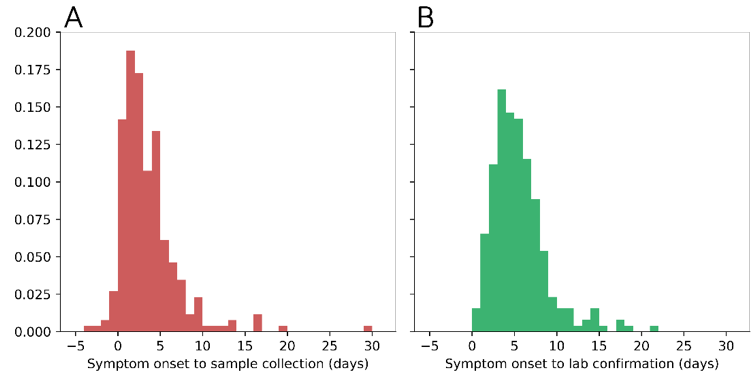


**Figure S2: Observed offspring distribution of COVID-19 cases in Karnataka, India.** Bars show observed frequency of the number of individuals infected by each case.

**[A]** Till 1 June (n=3404), **[B]** Till 13 June (n=6824), **[C]** Till 21 July (n=71068). See estimates in table S2.


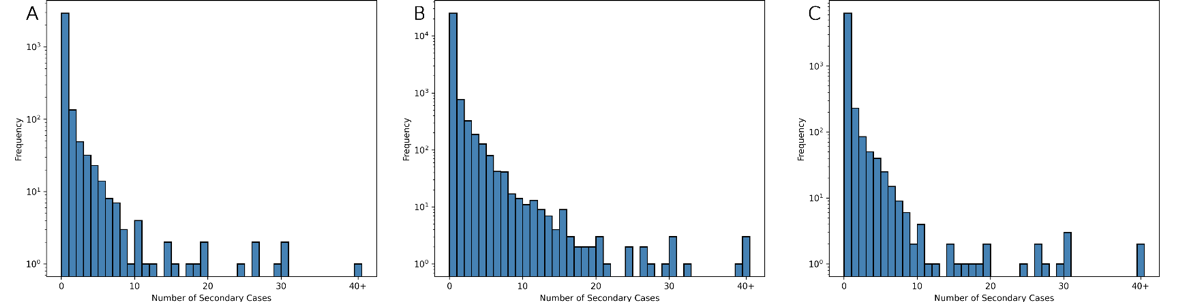


**References (for supporting materials)**

1 Health and Family Welfare Department, Government of Karnataka. Revised Quarantine and Testing Protocol for Primary-High Risk Contacts and Secondary - Low Risk Contacts. https://covid19.karnataka.gov.in/storage/pdf-files/cir-hws/Revised%20Quarantine%20and%20Testing%20Protocol%20for%20Primary-High%20Risk%20Contacts%20and%20Secondary%20-%20Low%20Risk%20Contacts.pdf.

2 Li R, Pei S, Chen B, *et al.* Substantial undocumented infection facilitates the rapid dissemination of novel coronavirus (SARS-CoV2). *Science* 2020; published online March 16. DOI:10.1126/science.abb3221.

3 Adam DC, Wu P, Wong JY, *et al.* Clustering and superspreading potential of SARS-CoV-2 infections in Hong Kong. *Nat Med* 2020; **26**: 1714–9.

4 Lau MSY, Grenfell B, Thomas M, Bryan M, Nelson K, Lopman B. Characterizing superspreading events and age-specific infectiousness of SARS-CoV-2 transmission in Georgia, USA. *Proc Natl Acad Sci* 2020; **117**: 22430–5.

5 Knight G, Leclerc QJ, Kucharski AJ. Analysis of SARS-CoV-2 transmission clusters and superspreading events. 2020; published online June 3.

6 Zou G. A Modified Poisson Regression Approach to Prospective Studies with Binary Data. *Am J Epidemiol* 2004; **159**: 702–6.

7 Public health criteria to adjust public health and social measures in the context of COVID-19: annex to considerations in adjusting public health and social measures in the context of COVID-19, 12 May 2020. World Health Organization, 2020.

8 Considerations in adjusting public health and social measures in the context of COVID-19: interim guidance, 16 April 2020. World Health Organization, 2020.

9 Indian Council of Medical Research (ICMR). Testing Strategy Updates for COVID-19 in India. https://www.icmr.gov.in/cteststrat.html (accessed Dec 3, 2020).
